# Cancer Screening Test Receipt — United States, 2018

**DOI:** 10.15585/mmwr.mm7002a1

**Published:** 2021-01-15

**Authors:** Susan A. Sabatino, Trevor D. Thompson, Mary C. White, Jean A. Shapiro, Janet de Moor, V. Paul Doria-Rose, Tainya Clarke, Lisa C. Richardson

**Affiliations:** ^1^Division of Cancer Prevention and Control, National Center for Chronic Disease Prevention and Health Promotion, CDC; ^2^Division of Cancer Control and Population Sciences, National Cancer Institute, Bethesda, Maryland; ^3^Division of Health Interview Statistics, National Center for Health Statistics, CDC.

Screening for breast cancer, cervical cancer, and colorectal cancer (CRC) reduces mortality from these cancers.* However, screening test receipt has been below national targets with disparities observed in certain populations (*1*,*2*). National Health Interview Survey (NHIS) data from 2018 were analyzed to estimate percentages of adults up to date with U.S. Preventive Services Task Force (USPSTF) screening recommendations. Screening test receipt remained below national Healthy People 2020 (HP2020) targets, although CRC test receipt neared the target. Disparities were evident, with particularly low test receipt among persons who were uninsured or did not have usual sources of care. Continued monitoring helps assess progress toward targets and could inform efforts to promote screening and reduce barriers for underserved populations.

Data from the 2018 NHIS, an annual survey of a nationally representative sample of the civilian, noninstitutionalized U.S. population,^†^ were used to examine up-to-date breast, cervical, and colorectal cancer screening test receipt per USPSTF recommendations. Information about tests was collected from one randomly selected adult per family (final sample adult response rate was 53.1%) (*3*). Respondents were asked whether they had ever received each test and when they received their most recent test. Respondents with a personal history of the cancer in question were excluded from analysis for that cancer type. Percentages with Korn-Graubard confidence intervals (*4*) are presented overall and by sociodemographic and health care access factors. Percentages of respondents who were up to date with screening were also age-standardized to the 2000 U.S. standard population, consistent with HP2020 cancer screening measures. NHIS-imputed income files were used. NHIS data from 2005, 2008, 2010, 2013, 2015, and 2018 were used to examine differences across years in percentages of persons who were up to date with screening, according to USPSTF recommendations in effect for each year. For 2018, “up-to-date” status was defined as receipt of the following: mammography within 2 years among women aged 50–74 years for breast cancer screening; Pap test within 3 years for women aged 21–65 years or Pap test plus human papillomavirus (HPV) test (co-testing) within 5 years for women aged 30–65 years for cervical cancer screening (among women without hysterectomy); and home blood stool or fecal immunochemical test (FIT) within 1 year; colonoscopy within 10 years; computed tomography (CT) colonography, or sigmoidoscopy within 5 years; or FIT-DNA test within 3 years among adults aged 50–75 years for CRC screening.

In August 2018, USPSTF added HPV testing alone as a cervical cancer screening option for women aged 30–65 years^§^; however, because this analysis used data collected beginning January 2018 regarding screening in the preceding 3–5 years, this option was not included. Wald F tests were used to test for any differences across years (treated categorically) and groups. Sample adult weights and design variables were used to account for the complex sample design. Estimates not meeting National Center for Health Statistics data presentation standards for proportions were suppressed (*4*). All analyses were performed using SAS (version 9.4; SAS Institute) and SUDAAN (version 11.0.3; RTI International).

Among women aged 50–74 years, 72.4% were up to date with mammography (age-standardized 72.3%) (Table 1), which is below the HP2020 target (81.1%). Lower test receipt was associated with having lower educational attainment and income, not having a usual source of care, and being uninsured or having only public health insurance coverage. Approximately 30%–40% of women without a usual source of care or health insurance coverage were up to date. Although the percentage of women up to date with mammography has not varied substantially by year (Figure), the absolute number of women who received a mammogram has increased. The estimated number of women tested (numerator) was 4,097,142 in 2005 and 5,558,224 in 2018, reflecting growth in the population of women aged 50–74 years (denominator) age-eligible for testing.

**TABLE 1:** Percentage of U.S. women age-eligible for screening who were up to date with breast and cervical cancer screening, by sociodemographic and access-to-care factors — United States, 2018

Characteristic	Breast cancer screening*		Cervical cancer screening^†^	
	No.	%^§^ (95% CI)	No.	%^§^ (95% CI)
**Overall**	5,311	72.4 (70.8–73.9)	7,732	82.9 (81.6–84.0)
**Age group, yrs** ^¶^				
21–30	—**	—**	1,717	75.8 (72.8–78.7)
31–40	—**	—**	1,989	90.1 (88.5–91.6)
41–50	—**	—**	1,590	87.9 (85.7–89.8)
51–65	—**	—**	2,436	79.5 (77.4–81.5)
50–64	3,229	71.5 (69.6–73.4)	—**	—**
65–74	2,082	74.3 (71.7–76.7)	—**	—**
P-value^††^	0.076		<0.001	
**Race**				
White	4,312	72.7 (71.0–74.3)	5,943	83.2 (81.9–84.5)
Black	625	72.9 (67.8–77.6)	1,038	87.1 (84.0–89.7)
AI/AN	52	—^§§^	102	73.6 (57.8–86.0)
Asian	210	70.5 (62.3–77.9)	460	75.8 (70.4–80.7)
Multiple race	108	65.3 (52.0–77.1)	173	77.5 (68.5–84.9)
P-value^††^	0.588		0.002	
**Ethnicity** ^¶¶^				
Non-Hispanic	4,768	72.6 (71.0–74.2)	6,475	83.2 (81.9–84.5)
Hispanic	543	70.7 (65.5–75.6)	1,257	81.4 (78.0–84.4)
Puerto Rican	64	79.8 (67.9–88.8)	127	81.1 (72.0–88.3)
Mexican/Mexican American	283	70.3 (62.9–77.1)	739	78.4 (73.5–82.7)
Central/South American	101	73.0 (59.2–84.1)	217	86.9 (79.8–92.2)
Other Hispanic	95	63.9 (51.3–75.2)	174	87.3 (80.1–92.7)
P-value^††^	0.471		0.283	
**Education**				
Less than high school	597	63.0 (57.7–68.1)	686	72.1 (67.3–76.7)
High school/GED	1,311	68.6 (65.5–71.5)	1,490	78.4 (75.5–81.2)
Some college	1,686	71.6 (68.9–74.2)	2,344	82.3 (80.2–84.2)
College degree	1,694	80.4 (78.1–82.7)	3,188	88.2 (86.5–89.8)
P-value^††^	<0.001		<0.001	
**Federal poverty threshold, %**				
≤138	1,060	58.6 (54.5–62.6)	1,677	73.7 (70.4–76.8)
>138–250	980	66.7 (62.6–70.6)	1,401	78.4 (75.3–81.4)
>250–400	1,030	72.1 (68.5–75.5)	1,556	84.3 (81.8–86.5)
>400	2,240	79.5 (77.3–81.6)	3,098	88.2 (86.7–89.7)
P-value^††^	<0.001		<0.001	
**Duration of U.S. residence, yrs** ^¶^				
≤10	51	—^§§^	303	65.0 (58.3–71.3)
>10	748	73.0 (68.4–77.2)	1,133	82.0 (78.9–84.8)
Born in United States	4,502	72.7 (71.1–74.3)	6,273	84.3 (83.0–85.5)
P-value^††^	0.028		<0.001	
**Sexual orientation**				
Gay or lesbian	63	—^§§^	124	64.7 (52.9–75.4)
Straight	5,118	72.6 (71.0–74.1)	7,288	83.4 (82.2–84.6)
Bisexual	24	—^§§^	171	79.0 (69.5–86.6)
Other	23	—^§§^	41	—^§§^
P-value^††^	0.304		0.007	
**Usual source of care**				
Yes	4,956	75.1 (73.6–76.6)	6,705	85.2 (84.0–86.4)
No	354	32.0 (26.1–38.4)	1,025	67.7 (63.9–71.3)
P-value^††^	<0.001		<0.001	
**Insurance**^¶,^ ***				
Private	3,305	77.2 (75.5–78.9)	5,302	86.4 (85.1–87.6)
Military	167	78.2 (70.2–85.0)	217	91.9 (86.6–95.6)
Public only	1,521	67.2 (64.2–70.2)	1,321	79.5 (76.4–82.4)
Uninsured	304	39.5 (32.8–46.5)	865	65.0 (60.6–69.1)
P-value^††^	<0.001		<0.001	

**Source:** National Center for Health Statistics, National Health Interview Survey, 2018.

**Abbreviations**: AI/AN = American Indian/Alaska Native; GED = General Educational Development certificate.

* Mammogram within preceding 2 years among women aged 50–74 years with no prior history of breast cancer.

^†^ For women without hysterectomy and with no prior history of cervical cancer, either Pap test within 3 years for women aged 21–65, or Pap test plus human papillomavirus (HPV) test (co-testing) within 5 years for women aged 30–65 years.

^§^ Percentages are weighted using National Health Interview Survey sample adult weights that adjust for the probability of selection, nonresponse, and poststratification. Poststratification adjustments for 2018 use population estimates derived from the 2010 Census by the U.S. Census Bureau.

^¶^ As of time of survey.

** Not estimated.

^††^ P-values from Wald F tests.

^§§^ Estimates suppressed because they did not meet National Center for Health Statistics reliability standards.

^¶¶^ P-value testing for differences between Hispanic persons and non-Hispanic persons. Hispanic subgroups are self-reported.

*** Insurance categorized hierarchically in order of categories listed.

**FIGURE:**
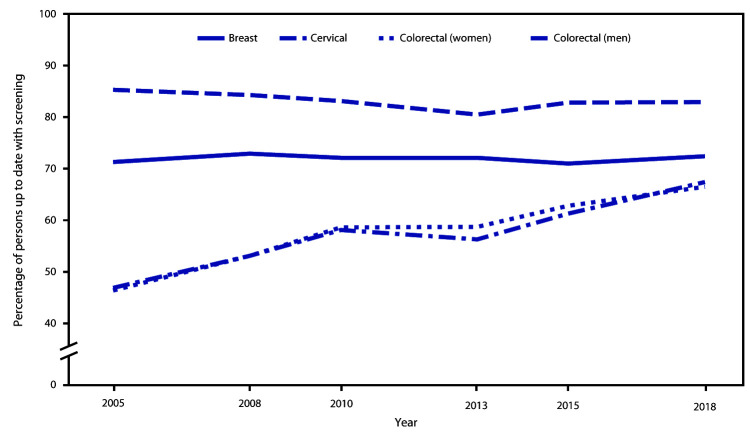
Percentage of adults up to date* with screening for breast, cervical, and colorectal cancers, by cancer type, sex, and year — United States, 2005–2018 * Up to date with U.S. Preventive Services Task Force screening recommendations in effect for each year defined as breast cancer: mammography within 2 years among women aged 50–74 years (all survey years); cervical cancer 2015–2018: Pap test within 3 years among women aged 21–65 years without hysterectomy, or Pap test plus human papillomavirus (HPV) test (co-testing) within 5 years among women aged 30–65 years without hysterectomy; cervical cancer before 2015: Pap test within 3 years among women aged 21–65 years without hysterectomy; colorectal cancer (CRC) 2018: home blood stool test within 1 year, sigmoidoscopy or computed tomography (CT) colonography within 5 years, colonoscopy within 10 years, or fecal immunochemical test (FIT)–DNA test within 3 years among adults aged 50–75 years; CRC 2010–2015: home blood stool test within 1 year, colonoscopy within 10 years, or sigmoidoscopy within 5 years with home blood stool test within 3 years among adults aged 50–75 years; CRC 2005–2008: home blood stool test within 1 year, colonoscopy within 10 years, or sigmoidoscopy within 5 years among adults aged 50–75 years.

Among women aged 21–65 years, 82.9% were up to date with cervical cancer screening (age-standardized 83.4%) (Table 1), which is below the HP2020 target (93.0%). Lower test receipt was associated with younger and older age groups, Asian race, lower educational attainment and income, shorter U.S. residence, gay or lesbian sexual orientation, no usual source of care, and being uninsured or having only public insurance coverage. Cervical cancer test receipt varied from 2005 to 2018 (Figure), with declines from 85.3% in 2005 to 80.5% in 2013, followed by an increase (82.9% in 2018).

Among adults aged 50–75 years, 66.9% were up to date with CRC testing (age-standardized 66.7%) (Table 2), nearing the HP2020 target (70.5%). Lower test receipt was associated with age 50–64 years, American Indian/Alaska Native or Asian race, Hispanic ethnicity, lower educational attainment or income, non-U.S. birthplace, no usual source of care, and non-military health insurance coverage or no insurance. Approximately 30% of those without a usual source of care or health insurance were up to date. Test receipt increased since 2005 (46.6%) (Figure).

**TABLE 2:** Percentage of U.S. adults aged 50–75 years who were up to date with colorectal cancer screening* — United States, 2018

Characteristic	Colorectal cancer screening	
	No.	%^†^ (95% CI)
Overall	10,595	66.9 (65.8–68.1)
**Age group, yrs** ^§^		
50–64	6,294	61.8 (60.2–63.3)
65–75	4,301	76.9 (75.4–78.4)
P-value^¶^	<0.001	
**Sex**		
Men	4,846	67.4 (65.8–69.0)
Women	5,749	66.5 (64.9–68.1)
P-value^¶^	0.437	
**Race**		
White	8,630	67.9 (66.6–69.2)
Black	1,197	65.3 (61.8–68.7)
AI/AN	116	54.7 (42.7–66.3)
Asian	432	58.1 (52.1–63.9)
Multiple race	201	66.9 (58.2–74.7)
P-value^¶^	0.007	
Ethnicity**		
Non-Hispanic	9,637	68.2 (67.0–69.4)
Hispanic	958	57.6 (53.4–61.7)
Puerto Rican	121	76.6 (67.2–84.4)
Mexican/Mexican American	513	52.3 (46.6–58.0)
Central/South American	173	57.7 (48.1–66.9)
Other Hispanic	151	63.4 (55.2–71.1)
P-value^¶^	<0.001	
**Education**		
Less than high school	1,132	54.2 (50.6–57.8)
High school/GED	2,704	63.5 (61.3–65.7)
Some college	3,218	67.7 (65.7–69.7)
College degree	3,499	73.5 (71.7–75.2)
P-value^¶^	<0.001	
**Federal poverty threshold, %**		
≤138	1,881	56.9 (53.9–60.0)
>138–250	1,924	59.7 (56.8–62.7)
>250–400	2,053	66.3 (63.4–69.0)
>400	4,737	72.7 (71.0–74.3)
P-value^¶^	<0.001	
**Duration of U.S. residence^¶^**		
≤10 yrs	81	32.8 (21.5–45.8)
>10 yrs	1,384	58.6 (55.3–61.8)
Born in U.S.	9,113	69.2 (68.0–70.4)
P-value^¶^	<0.001	
**Sexual orientation**		
Gay or lesbian	199	75.3 (67.2–82.3)
Straight	10,140	66.9 (65.8–68.1)
Bisexual	44	—^††^
Other	44	—^††^
P-value^¶^	0.118	
**Usual source of care**		
Yes	9,739	70.2 (69.0–71.3)
No	856	29.4 (25.5–33.5)
P-value^§^	<0.001	
**Insurance** ^§,§§^		
Private	6,488	69.0 (67.5–70.4)
Military	631	80.6 (76.7–84.1)
Public only	2,812	68.2 (66.1–70.4)
Uninsured	640	30.2 (25.5–35.1)
P-value^¶^	<0.001	

**Source:** National Center for Health Statistics, National Health Interview Survey, 2018.

**Abbreviations:** AI/AN = American Indian/Alaska Native; GED = General Educational Development.

* Among respondents aged 50–75 years with no prior history of colorectal cancer, home blood stool or fecal immunochemical test (FIT) within 1 year, colonoscopy in past 10 years, computed tomography (CT) colonography in past 5 years, sigmoidoscopy in past 5 years, or FIT-DNA test in past 3 years.

^†^ Weighted using National Health Interview Survey sample adult weights that adjust for the probability of selection, nonresponse, and post-stratification. Post-stratification adjustments for 2018 use population estimates derived from the 2010 Census by the U.S. Census Bureau.

^§^ As of time of survey.

^¶^ P-values from Wald F tests.

** P-value testing for differences between Hispanic persons and non-Hispanic persons. Hispanic subgroups are self-reported.

^††^ Estimates suppressed because they did not meet National Center for Health Statistics reliability standards.

^§§^ Insurance categorized hierarchically in order of categories listed.

## Discussion

In 2018, receipt of screening tests for breast, cervical, and colorectal cancers was below national HP2020 targets. CRC test receipt increased after 2005 and neared the target in 2018, whereas breast and cervical cancer test receipt remained below targets with little change over this period. Test receipt varied across groups. As was also found in previous reports, testing for all three cancers decreased with decreasing educational attainment and income (*1*,*2*). Cervical cancer test receipt differed by sexual orientation, CRC test receipt varied by ethnicity, and both differed by age, race, and duration of U.S. residence. Information about lower test receipt in some groups might help inform targeted efforts to promote screening and reduce disparities. Lower test receipt in the youngest age groups for cervical cancer and CRC screening might, in part, reflect the transition of persons who previously did not meet screening criteria.

The lowest percentages of breast cancer and CRC screening test receipt were among respondents who lacked a usual source of care (32.0% and 29.4% for breast cancer and CRC screening, respectively) or health insurance coverage (39.5% and 30.2% for breast cancer and CRC screening, respectively); the largest disparities on the basis of these characteristics were for breast cancer and CRC screening. Most persons in these groups were not up to date with breast cancer or CRC tests. These large disparities have persisted for years (*1*,*2*,*5*,*6*). The number of persons without health insurance has declined in recent years (*7*). However, among those lacking insurance or a usual source of care, most were not up to date with USPSTF breast cancer and CRC screening recommendations. CDC’s National Breast and Cervical Cancer Early Detection Program provides low-income, uninsured, and underinsured women access to breast and cervical cancer screening and diagnostic services.^¶^ The Colorectal Cancer Control Program supports implementation of evidence-based interventions and supporting strategies in health systems to increase screening use.** Even among those with health insurance coverage, some groups might be farther below targets than others. For example, approximately 77%–78% of women with private or military insurance were up to date with USPSTF breast cancer screening recommendations, nearing the HP2020 target of 81.1%, compared with 67% of women with only public insurance. Of note, HP2020 determined targets based on population totals rather than specific groups.

The findings reported reflect receipt of tests within recommended screening intervals. They do not reflect test overuse, screening quality, or adequacy of follow-up. For example, positive results on CRC screening stool tests need follow-up colonoscopy to complete evaluation, and problems in CRC screening quality exist (*8*,*9*).

The findings in this report are subject to at least five limitations. First, data are self-reported and potentially subject to social desirability and recall bias. Second, survey questions about tests have changed over time. Third, the 2018 sample adult response rate was 53%, and nonresponse bias might exist despite survey weight adjustments; response rates for earlier years have been published (*3*). Fourth, because of limited sample sizes, estimates could not be generated for all groups. Finally, percentages might include tests performed for diagnostic purposes. NHIS data from 2018 include self-reported reasons for mammograms but not for cervical cancer tests or the CRC screening measure. Among women who received a mammogram within 2 years in the current analysis, 95% reported that it was part of a “routine exam.” A study of CRC tests (*10*) also suggested that a majority of respondents reported that tests were performed for screening. Consistent with HP2020 measures^††^ and previous reports (*1*,*2*,*5*,*6*,*10*), the current analysis included all tests because those receiving diagnostic tests might be considered screened in effect and therefore up to date with screening recommendations.

Continued monitoring can help assess whether national screening targets are achieved, and inform efforts that promote screening test receipt as recommended and reduce barriers for underserved populations to eliminate disparities. To promote screening for these three cancers, the Community Preventive Services Task Force recommends evidence-based interventions that increase client demand for, access to, and provider delivery of screening services.^§§^ The Task Force noted that evidence-based interventions can be selected and adapted to meet the needs of communities and specific populations and can be combined to address multiple barriers, potentially at multiple levels. Resources are available to help identify, implement, and evaluate evidence-based approaches through The Community Guide, Evidence-Based Cancer Control Programs^¶¶^ and Cancer Control P.L.A.N.E.T. (Plan, Link, Act, Network with Evidence-based Tools).***

**Summary**

**What is already known about this topic?**

Receipt of screening for breast cancer, cervical cancer, and colorectal cancer (CRC) is below national targets. Large population disparities in screening receipt exist.

**What is added by this report?**

In 2018, receipt of screening tests for breast and cervical cancers remained below Healthy People 2020 targets, with little change since 2005. CRC screening receipt increased in recent years and has neared the target (70.5%). Screening test receipt was low among persons without health insurance coverage or a usual source of care.

**What are the implications for public health practice?**

Continued monitoring of screening rates can help assess whether national screening targets are achieved. Information about test receipt might help inform efforts that promote screening test use as recommended and reduce barriers for underserved populations to eliminate disparities.

## Footnotes

* https://uspreventiveservicestaskforce.org/uspstf/recommendation/breast-cancer-screening#bootstrap-panel--5; https://uspreventiveservicestaskforce.org/uspstf/recommendation/cervical-cancer-screening#bootstrap-panel--8; https://uspreventiveservicestaskforce.org/uspstf/recommendation/colorectal-cancer-screening#bootstrap-panel--5.

† https://www.cdc.gov/nchs/nhis/nhis_2018_data_release.htm.

§ https://uspreventiveservicestaskforce.org/uspstf/recommendation/cervical-cancer-screening.

¶ https://www.cdc.gov/cancer/nbccedp/.

** https://www.cdc.gov/cancer/crccp/about.htm.

†† https://www.healthypeople.gov/2020/data-search/Search-the-Data#topic-area=3513;

§§ https://www.thecommunityguide.org/.

¶¶ https://ebccp.cancercontrol.cancer.gov/.

*** https://cancercontrolplanet.cancer.gov/planet/.

## References

[1] White A, Thompson TD, White MC, Cancer screening test use—United States, 2015. MMWR Morb Mortal Wkly Rep 2017;66:201–6. 10.15585/mmwr.mm6608a1 28253225 PMC5657895

[2] Sabatino SA, White MC, Thompson TD, Klabunde CN; CDC. Cancer screening test use—United States, 2013. MMWR Morb Mortal Wkly Rep 2015;64:464–8. 25950253 PMC4584551

[3] CDC. National Health Interview Survey: survey description. Hyattsville, MD: US Department of Health and Human Services, CDC, National Center for Health Statistics; 2019. ftp://ftp.cdc.gov/pub/Health_Statistics/NCHS/Dataset_Documentation/NHIS/2018/srvydesc.pdf.

[4] Parker JD, Talih M, Malec DJ, National Center for Health Statistics data presentation standards for proportions. Vital Health Stat 2 2017;2:1–22. 30248016

[5] Shapiro JA, Klabunde CN, Thompson TD, Nadel MR, Seeff LC, White A. Patterns of colorectal cancer test use, including CT colonography, in the 2010 National Health Interview Survey. Cancer Epidemiol Biomarkers Prev 2012;21:895–904. 10.1158/1055-9965.EPI-12-0192 22490320 PMC4489134

[6] Swan J, Breen N, Coates RJ, Rimer BK, Lee NC. Progress in cancer screening practices in the United States: results from the 2000 National Health Interview Survey. Cancer 2003;97:1528–40. 10.1002/cncr.11208 12627518

[7] CDC. National Health Interview Survey: long-term trends in health insurance. Atlanta, GA: US Department of Health and Human Services, CDC: 2019. https://www.cdc.gov/nchs/data/nhis/health_insurance/TrendHealthInsurance1968_2018.pdf.

[8] Butterly LF, Nadel MR, Anderson JC, Impact of colonoscopy bowel preparation quality on follow-up interval recommendations for average-risk patients with normal screening colonoscopies. J Clin Gastroenterol 2020;54:356–64. 10.1097/MCG.0000000000001115 30106836 PMC6374206

[9] Nadel MR, Royalty J, Joseph D, Variations in screening quality in a federal colorectal cancer screening program for the uninsured. Prev Chronic Dis 2019;16:E67. 10.5888/pcd16.180452 31146803 PMC6549419

[10] de Moor JS, Cohen RA, Shapiro JA, Colorectal cancer screening in the United States: trends from 2008 to 2015 and variation by health insurance coverage. Prev Med 2018;112:199–206. 10.1016/j.ypmed.2018.05.001 29729288 PMC6202023

